# Supplementary material for: Differential Expression and Function of SVIP in Breast Cancer Cell Lines and In Silico Analysis of Its Expression and Prognostic Potential in Human Breast Cancer
Source: Cells. 2023 May 11;12(10):1362. doi: 10.3390/cells12101362 (PMC10216740; doi:10.3390/cells12101362)
Supplement: Supplementary file 1 [file cells-12-01362-s001.zip › cells-2322815-supplementary.pdf]

Figure S1

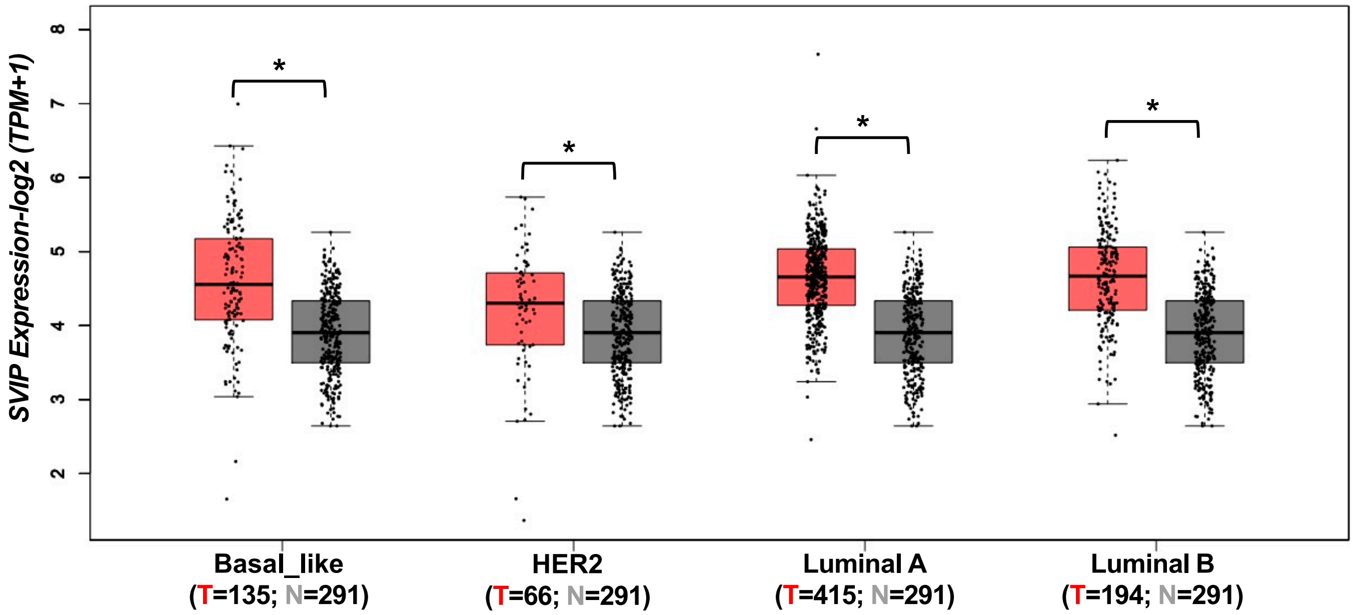

Figure S2

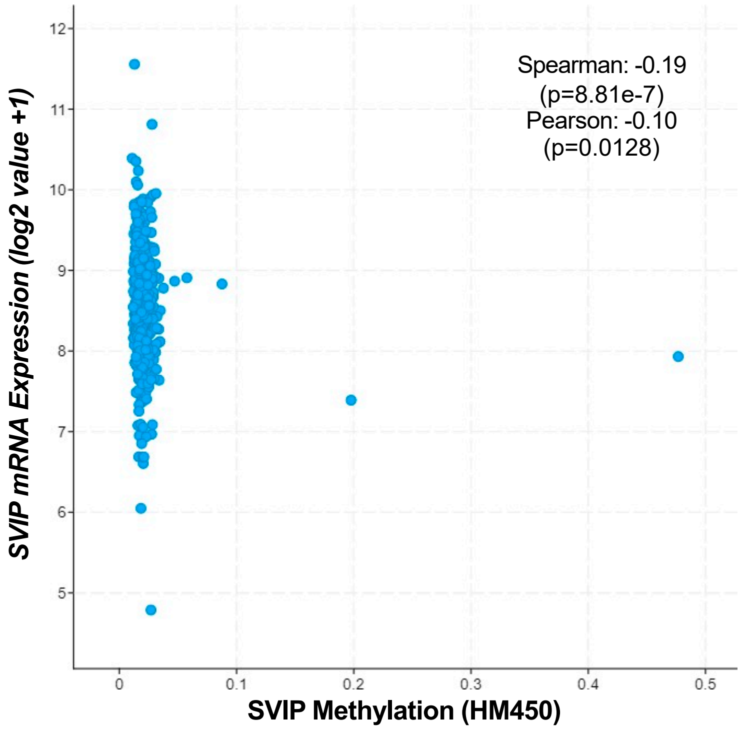

Figure S3

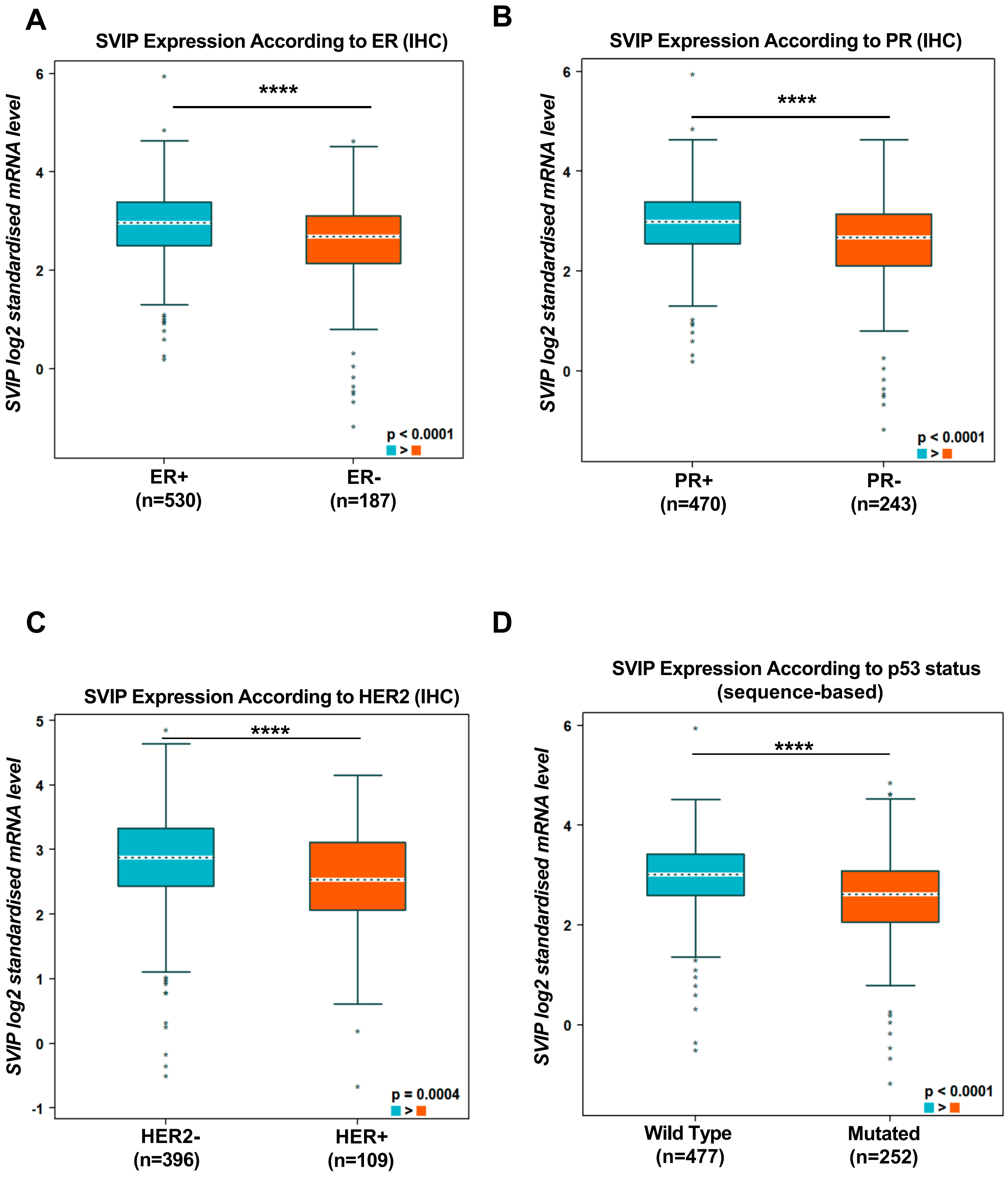
A
SVIP Expression According to ER (IHC)
SVIP log2 standardised mRNA level
****
p < 0.0001
ER+
(n=530)
ER-
(n=187)
B
SVIP Expression According to PR (IHC)
SVIP log2 standardised mRNA level
****
p < 0.0001
PR+
(n=470)
PR-
(n=243)
C
SVIP Expression According to HER2 (IHC)
SVIP log2 standardised mRNA level
****
p = 0.0004
HER2-
(n=396)
HER+
(n=109)
D
SVIP Expression According to p53 status
(sequence-based)
SVIP log2 standardised mRNA level
****
p < 0.0001
Wild Type
(n=477)
Mutated
(n=252)

Figure S4

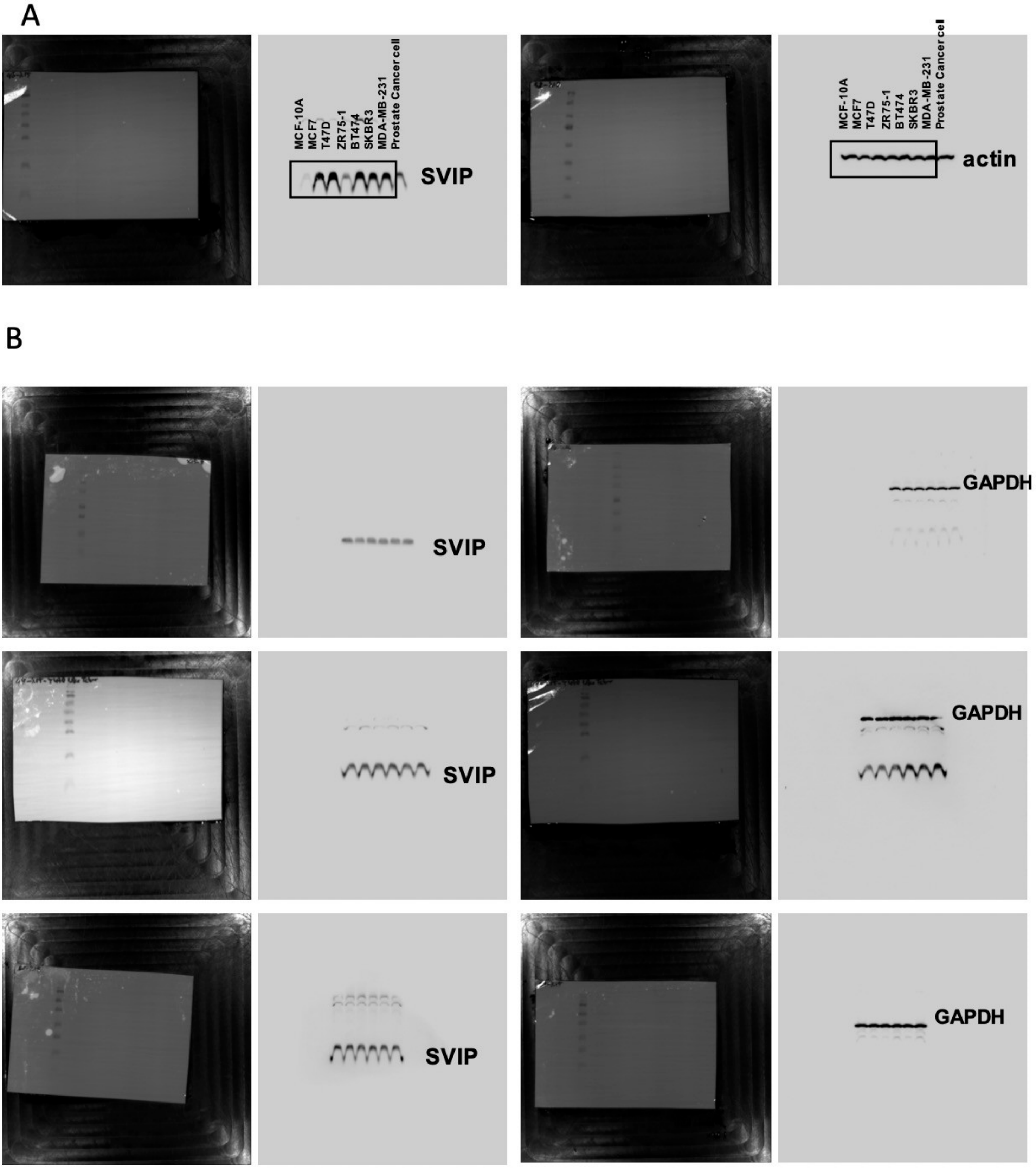
A
MCF-10A
MCF7
T47D
ZR75-1
BT474
SKBR3
MDA-MB-231
Prostate Cancer cell
SVIP
actin
B
SVIP
GAPDH
SVIP
GAPDH
SVIP
GAPDH

C

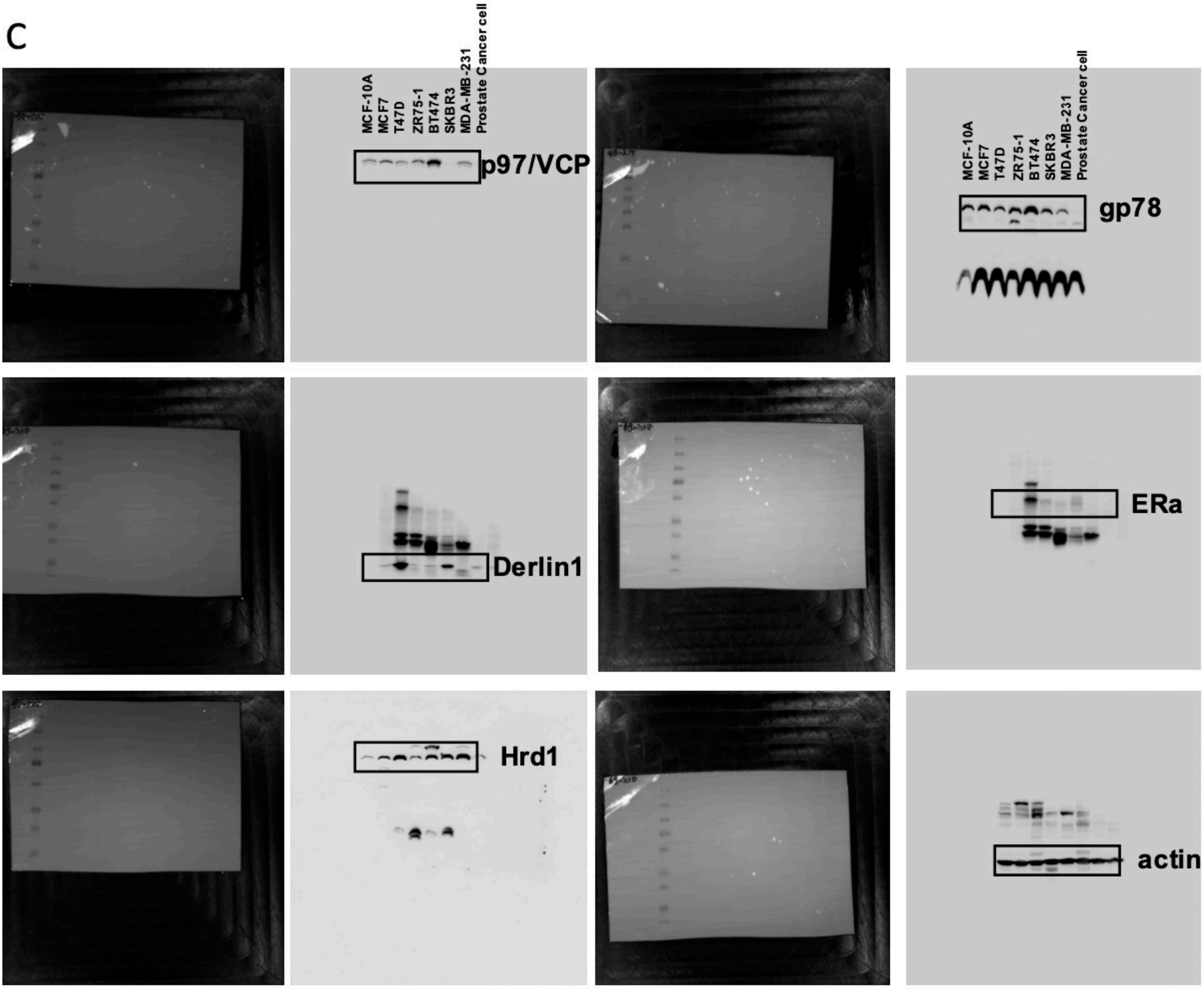
MCF-10A
MCF7
T47D
ZR75-1
BT474
SKBR3
MDA-MB-231
Prostate Cancer cell
p97/VCP
gp78
Derlin1
ERa
Hrd1
actin

D

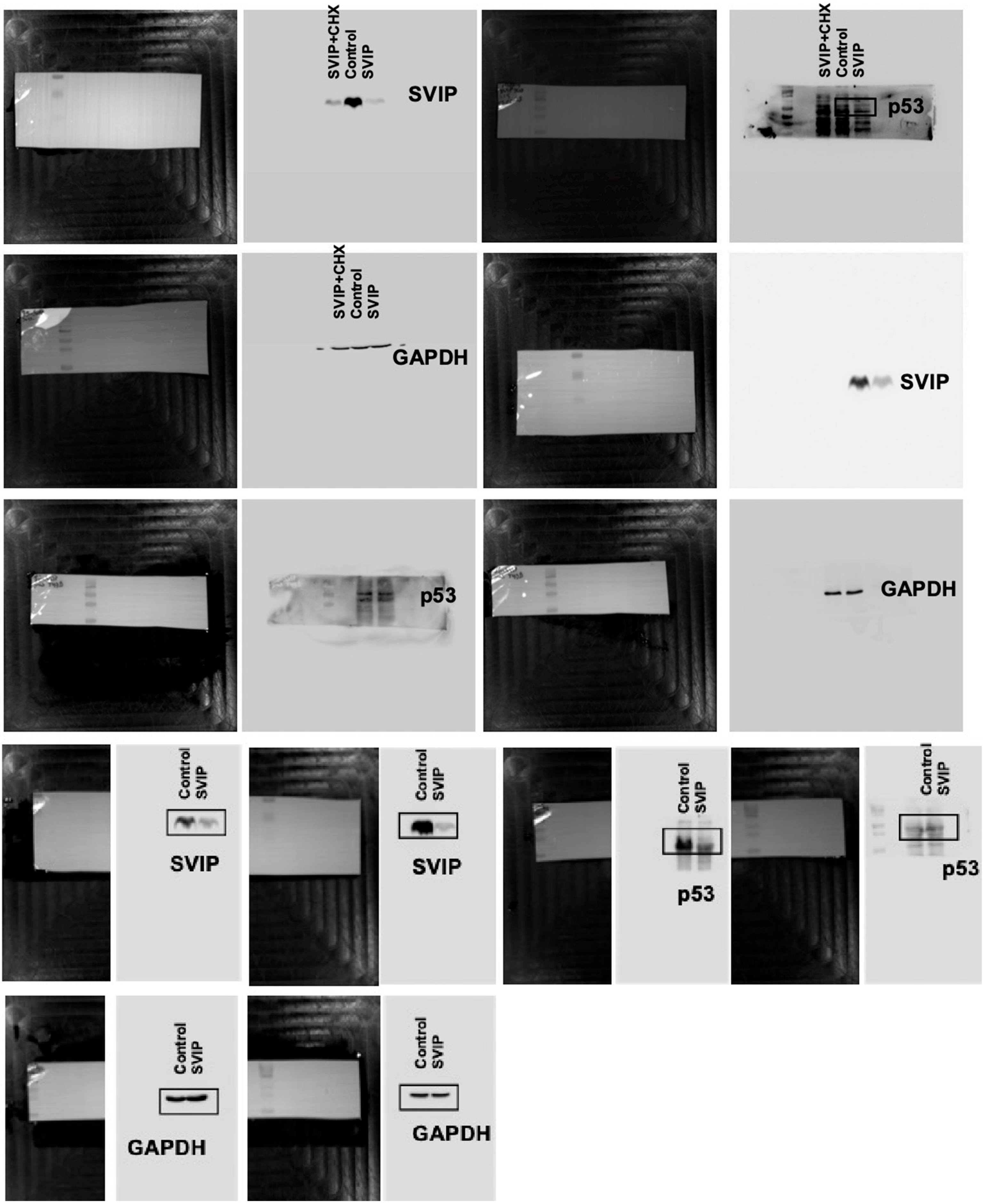
SVIP+CHX
Control
SVIP
SVIP
SVIP+CHX
Control
SVIP
p53
SVIP+CHX
Control
SVIP
GAPDH
SVIP
p53
GAPDH
Control
SVIP
SVIP
Control
SVIP
SVIP
Control
SVIP
p53
Control
SVIP
p53
Control
SVIP
GAPDH
Control
SVIP
GAPDH

E

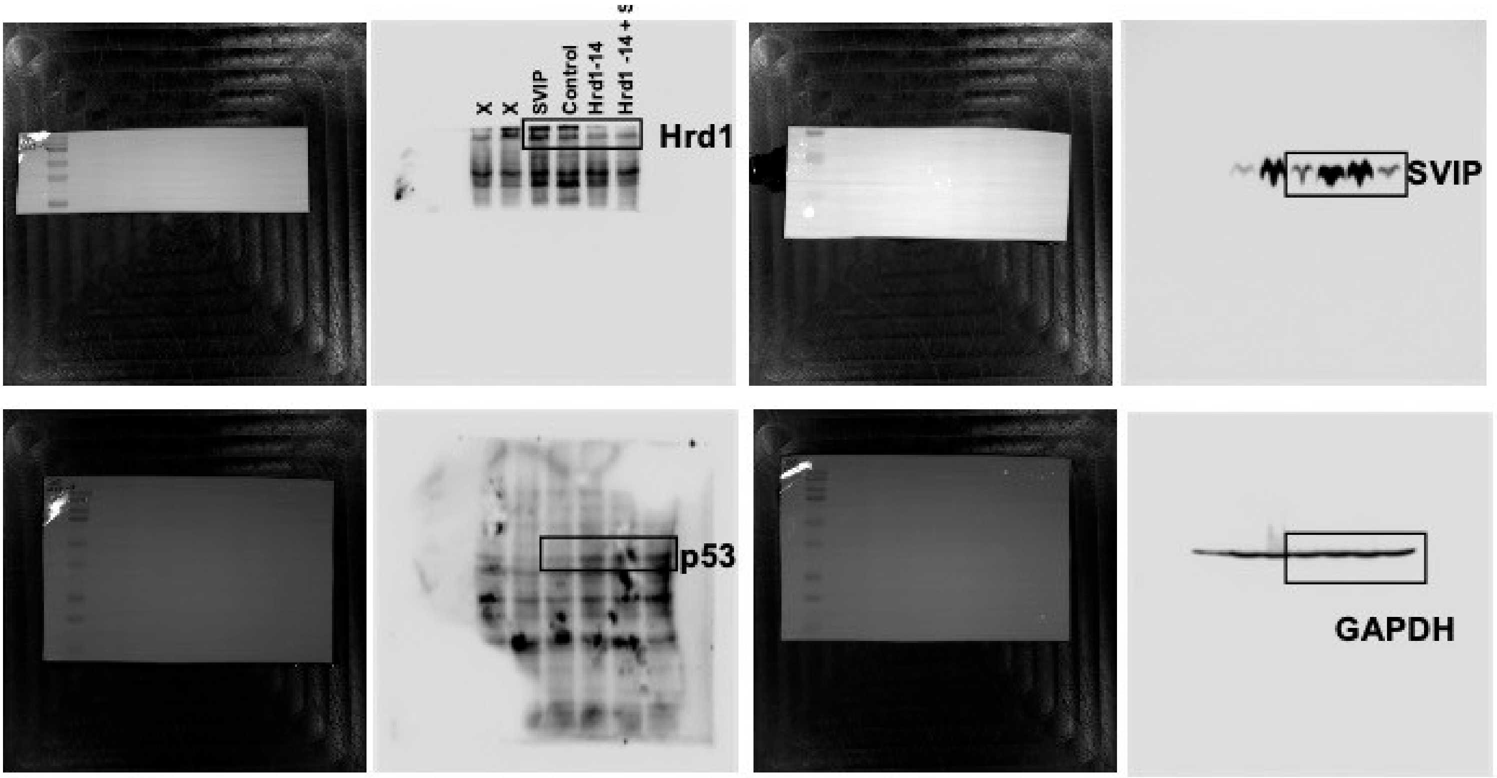

Supplementary Fig. S1. SVIP expression in breast cancer different subtypes and normal tissues (GEPIA). In the SVIP expression levels analysis using GEPIA, the threshold included expression fold change (Log2FC) ≥ 1 between breast cancer subtype and normal tissues, * $p < 0.01$. T = tumor tissues. N = normal tissues, NUM: number.

Supplementary Fig. S2. Correlation of SVIP mRNA expression and methylation (cBioPortal). (Spearman=-019, p=8.81e-7; Pearson=-0.10, *p*= 0.0128)

Supplementary Fig. S3. UALCAN, GEPIA, and bc-GenExMiner v4.8 portals analysis of breast cancer samples from the TCGA and GTEx datasets **A)** The mRNA expression level of SVIP in breast cancer patients with ER (−) and ER (+) (****, $p < 0.0001$) (bc-GenExMiner software). **B)** The mRNA expression level of SVIP in breast cancer patients with PR (−) and PR (+) (****, $p < 0.0001$) (bc-GenExMiner software).**)** **C)** The mRNA expression level of SVIP in breast cancer patients with HER2 (−) and HER2 (+) (***, $p = 0.0004$) (bc-GenExMiner software). **D)** The mRNA expression level of SVIP in breast cancer patients with p53 wild type and p53 mutated (****, $p < 0.0001$) (bc-GenExMiner software). IHC: Immunohistochemistry

Supplementary Fig. S4.. Original Blots of immunoblotting experiments
